# DANTE preparation for black-blood coronary wall imaging

**DOI:** 10.1186/1532-429X-15-S1-P237

**Published:** 2013-01-30

**Authors:** Meihan Wang, Christopher M Kramer, Craig H Meyer

**Affiliations:** 1 Biomedical Engineering, University of Virginia, Charlottesville, VA, USA; 2 Medicine, University of Virginia, Charlottesville, VA, USA; 3 Radiology, University of Virginia, Charlottesville, VA, USA

## Background

Double inversion recovery (DIR) pre-pulses combined with ECG gating are often used [1] in black blood imaging. However, the inversion time is often too long. Also, the blood suppression is often insufficient in multi-slice studies. DANTE is a novel black-blood technique that has been shown to maintain SNR and CNR similar to single-slice DIR in ungated carotid artery studies [2] and is compatible with multi-slice acquisition. In this study, we combined DANTE pulses with gating and tested the performance for coronary wall imaging.

## Methods

Experiments were performed on a Siemens 3T Tim Trio scanner with a 32 channel coil array. Four volunteers (age 26-29) were tested with a multi-slice spiral gradient echo sequence with ECG gating. The sequence consisted of a 5ms spectral-spatial excitation pulse followed by a 16ms constant density spiral readout gradient. FOV = 240-340mm, 14-20 interleaves, 3 slices, each with 5mm, inplane resolution 0.7-0.9mm. DANTE prepulses were placed before the imaging readout with spacing = 0.6ms and flip angle = 7. A similar sequence with double inversion pre-pulses was used for comparison.

## Results

**Figure 1:**
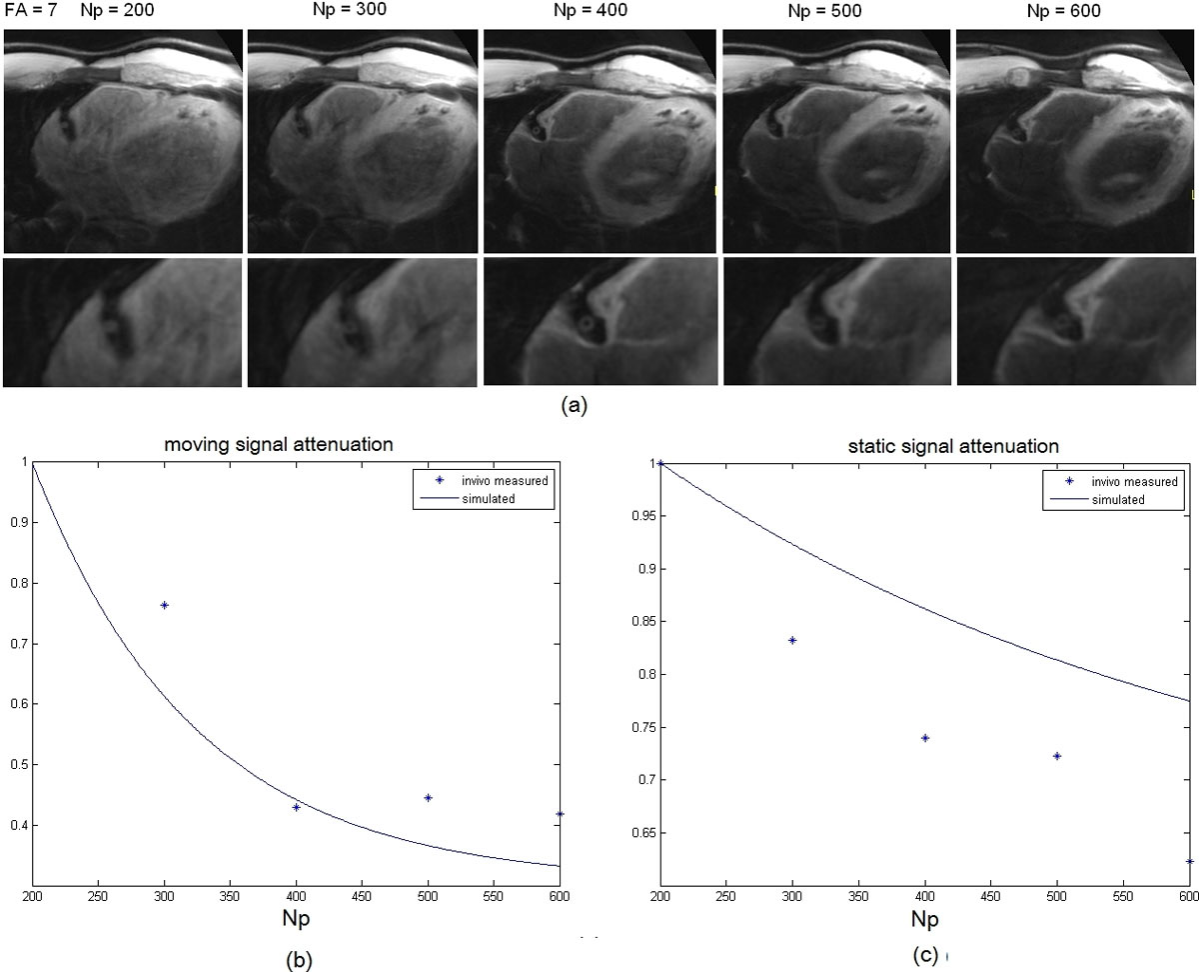
(a) RCA wall images acquired with DANTE preparation, with number of pulses = 200, 300, 400, 500 and 600 and flip angle = 7. (b) Simulated and experimental signal attenuation of moving spin using the DANTE preparation. (c) Simulated and experimental signal attenuation of static spin using the DANTE preparation.

As shown in Fig. 1, the blood suppression improved as the number of pulses (Np) increased from 200 to 600, but there was increasing attenuation of the wall signal. The best contrast between wall and lumen was achieved when = 400-500. There was good agreement between the measured and simulated blood suppression, as shown in Fig. 1(b). Fig.1(c) shows that the vessel wall signal experiences extra attenuation because of motion.

**Figure 2:**
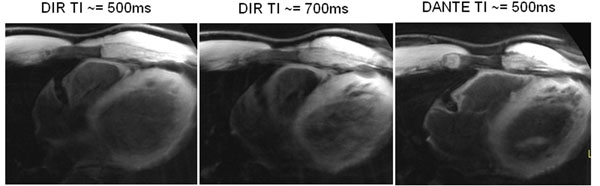
Cross-sectional view of RCA with (a) DIR, short TI (b) DIR, long TI and (c) DANTE.

For some subjects, the rest period occurred before mid-diastole, which left unsuppressed blood when using DIR, as shown in Fig. 2(a). Figure 2(b) shows the result using DIR with a longer inversion time. The image has better blood suppression, but the coronary wall is severely blurred by motion of the RCA. As shown in Fig. 2(c), DANTE pulses enable a better balance between nulling blood and reducing motion blur.

The mean SNR and CNR of the coronary wall was 14.9+/-3.8 and 5.5+/-3.1 for images with DANTE pulses, and 16.7+/-3.7 and 5.1+/-3.0 for DIR prepared images. DIR achieved a slightly higher SNR but lower CNR because of unsuppressed blood induced by the short TI (p<0.05).

## Conclusions

We have developed a cardiac-gated DANTE black blood technique for coronary artery imaging. DANTE pulses give comparable SNR to double inversion recovery, but with more flexible imaging. This method can be adapted for other black blood cardiac applications.

## Funding

This study was funded by NIH R01 HL079110 and Siemens Medical Solutions.

